# Variation for N Uptake System in Maize: Genotypic Response to N Supply

**DOI:** 10.3389/fpls.2015.00936

**Published:** 2015-11-09

**Authors:** Trevor Garnett, Darren Plett, Vanessa Conn, Simon Conn, Huwaida Rabie, J. Antoni Rafalski, Kanwarpal Dhugga, Mark A. Tester, Brent N. Kaiser

**Affiliations:** ^1^Australian Centre for Plant Functional Genomics, School of Agriculture Food and Wine, University of AdelaideAdelaide, SA, Australia; ^2^School of Agriculture, Food and Wine, University of AdelaideAdelaide, SA, Australia; ^3^Mathematics Department, Bethlehem UniversityBethlehem, Palestine; ^4^DuPont Crop GeneticsWilmington, DE, USA; ^5^Genetic Discovery, DuPont PioneerJohnston, IA, USA

**Keywords:** N, nitrate, ammonium, nitrogen use efficiency, NUE, uptake, *Zea mays*

## Abstract

An understanding of the adaptations made by plants in their nitrogen (N) uptake systems in response to reduced N supply is important to the development of cereals with enhanced N uptake efficiency (NUpE). Twenty seven diverse genotypes of maize (*Zea mays*, L.) were grown in hydroponics for 3 weeks with limiting or adequate N supply. Genotypic response to N was assessed on the basis of biomass characteristics and the activities of the nitrate (${\text{NO}}_{3}^{-}$) and ammonium (${\text{NH}}_{4}^{+}$) high-affinity transport systems. Genotypes differed greatly for the ability to maintain biomass with reduced N. Although, the N response in underlying biomass and N transport related characteristics was less than that for biomass, there were clear relationships, most importantly, lines that maintained biomass at reduced N maintained net N uptake with no change in size of the root relative to the shoot. The root uptake capacity for both ${\text{NO}}_{3}^{-}$ and ${\text{NH}}_{4}^{+}$ increased with reduced N. Transcript levels of putative ${\text{NO}}_{3}^{-}$ and ${\text{NH}}_{4}^{+}$ transporter genes in the root tissue of a subset of the genotypes revealed that predominately ZmNRT2 transcript levels responded to N treatments. The correlation between the ratio of transcripts of *ZmNRT2.2* between the two N levels and a genotype's ability to maintain biomass with reduced N suggests a role for these transporters in enhancing NUpE. The observed variation in the ability to capture N at low N provides scope for both improving NUpE in maize and also to better understand the N uptake system in cereals.

## Introduction

Over 100 million tons of nitrogen (N) fertilizer are applied worldwide annually in an effort to maximize crop yields with more than half being used to grow cereals (FAO, 2008). Nitrogen use efficiency (NUE, calculated as [grain N harvested)/(supplied N)] of cereals is estimated at only 33% (Raun and Johnson, 1999). Underutilized fertilizer represents an unnecessary expense for farmers; it also leads to a range of environmental problems including pollution of groundwater, rivers and oceans as well as being a significant contributor to greenhouse gas emissions (Davidson, 2009).

Maize (*Zea mays*, L.) is currently the crop with highest production among all crops and is also amongst those with the greatest demands for N fertilizer (Sivasankar et al., 2012). Consequently, any improvements made in N fertilizer use will bring significant monetary and environmental benefits. Although, improved fertilization practices may lead to enhanced NUE (Keeney, 1982), a complementary approach is the improvement of germplasm, either by increasing the N uptake efficiency (NUpE) or the N utilization efficiency (NUtE) (Good et al., 2004). For cereals, the relative importance of NUpE or NUtE to the overall NUE appears to depend on the level of N supply. Moll et al. (1982) found that when maize was supplied with high N, NUpE was more important, whereas at low N supply it was NUtE; the same conclusion was reached with wheat (Ortiz-Monasterio et al., 1997). Generally, at less than 40%, the NUpE of cereals is poor (Peoples et al., 1995; Sylvester-Bradley and Kindred, 2009) which indicates considerable scope for the improvement of this component of N use.

Regardless of the form of applied N fertilizer, nitrate (${\text{NO}}_{3}^{-}$) is the dominant source of N in agricultural soils and thus the major component of N uptake by cereal crops (Wolt, 1994; Miller et al., 2007). However, although generally present at only 10% of the ${\text{NO}}_{3}^{-}$ concentration, ammonium (${\text{NH}}_{4}^{+}$) can still make a significant contribution to the overall plant N budget (Wolt, 1994; Miller et al., 2007). As ${\text{NO}}_{3}^{-}$ is highly mobile in the soil, ${\text{NO}}_{3}^{-}$ uptake is dependent to a greater degree on the uptake capacity of the roots than on root morphology (Burns, 1980; Robinson and Rorison, 1983). Although, ${\text{NH}}_{4}^{+}$ is less readily mobile in the soil than ${\text{NO}}_{3}^{-}$, ${\text{NH}}_{4}^{+}$ uptake capacity of the roots is still more important than it is for immobile nutrients such as phosphorous (Clarkson, 1985). Little is known with regards to genetic variability in maize roots in terms of ${\text{NO}}_{3}^{-}$ or ${\text{NH}}_{4}^{+}$ uptake capacity. Field based studies found differences in N uptake within a selection of hybrids (Pollmer et al., 1979). Pace and Mcclure (1986) measured ${\text{NO}}_{3}^{-}$ uptake capacity in 85 maize genotypes and found considerable variability. Such variability could be useful both in terms of making selections for elite germplasm, and also in providing a better understanding of the N uptake process in maize and the way in which it could be enhanced. However, this work was based on 5 day-old seedlings grown in the dark, so may be of limited utility.

Since the Pace and McClure study the identification of transport proteins involved in the uptake of both ${\text{NO}}_{3}^{-}$ and ${\text{NH}}_{4}^{+}$ by roots and some elucidation of the regulation of these proteins has resulted in a significantly improved understanding of the processes underlying the N uptake capacity of roots. For ${\text{NO}}_{3}^{-}$, plant roots have both a low affinity transport system (LATS) and a high affinity transport system (HATS) (Siddiqi et al., 1990; Kronzucker et al., 1995). Nitrate uptake in the LATS range involves predominantly NRT1 proteins whereas NRT2 proteins contribute most to the HATS system activity (Okamoto et al., 2003; Tsay et al., 2007). The ${\text{NH}}_{4}^{+}$ transport systems are not well characterized but similarly comprise a HATS and LATS with AMTs being the proteins predominantly responsible for uptake (von Wirén et al., 2000; Kaiser et al., 2002; Ludewig et al., 2007; Gu et al., 2013). Despite an improved understanding of N uptake by plant roots, knowledge of the transport systems involved in meeting N requirements in response to N demand and supply remains limited. A recent study in maize by Garnett et al. (2013) indicated that for ${\text{NO}}_{3}^{-}$ it was the HATS, specifically NRT2.1 and NRT2.2, that were chiefly responsible for responding to N supply and demand; this was found to be the case even in plants grown with ${\text{NO}}_{3}^{-}$ levels within the LATS concentration range.

In order to evaluate the extent of genetic variation in response to N supply in maize, the current study involved the quantification of differences in growth and uptake capacity for both ${\text{NO}}_{3}^{-}$ and ${\text{NH}}_{4}^{+}$ for a diverse range of maize inbred genotypes. Plants were grown in hydroponics with sufficient or reduced N for 3 weeks before being harvested, and the uptake capacity of the ${\text{NO}}_{3}^{-}$ and ${\text{NH}}_{4}^{+}$ high-affinity transport systems (HATS) was measured using short term measurements of unidirectional fluxes of ^15^N labeled ${\text{NO}}_{3}^{-}$ and ${\text{NH}}_{4}^{+}$ (Garnett et al., 2013). As a means of better understanding the differences in uptake capacity, and clarifying which transporters are responsible for the observed uptake capacity, the transcriptional response of genes encoding putative maize ${\text{NO}}_{3}^{-}$ transporters identified by Plett et al. (2010), together with two high affinity ${\text{NH}}_{4}^{+}$ transporters (Gu et al., 2013), was also determined in a subset of the genotypes using quantitative real time PCR.

## Materials and methods

### Plant material

Maize (*Zea mays*, L.) inbred genotypes were chosen to represent a range of heterotic groups. The backgrounds are presented in Supplementary Table S1. Genotypes were sourced from either the USDA, ARS, North Central Regional Plant Introduction Station at Iowa State University, Ames, Iowa, or from Pioneer Hi-Bred, Johnston, Iowa.

### Plant growth

A total of seven experiments were carried out with groups of five genotypes in each experiment with B73 as a check genotype common to all experiments. Seeds were first rinsed several times in reverse osmosis purified (RO) water and then aerated overnight prior to being spread on 2 layers of Whatman No. 42 filter paper in petri dishes and placed in an incubator at 28°C for 72 h. The seeds were kept moist with RO water. Seedlings were transferred to one of two, 700 l ebb-and-flow hydroponic systems as described in Garnett et al. (2013). The hydroponic system was situated in a controlled environment room with 14/10-h 25°C/20°C day/night cycle at a flux density at canopy level of approximately 500 μm.m^−2^.s^−1^. The nutrient solution was a modified Johnson's solution (Johnson et al., 1957) containing either (in mM) 0.5 NO_3_-N, 0.8 K, 0.1 Ca, 0.5 Mg, 1 S, and 0.5 P for the 0.5 mM ${\text{NO}}_{3}^{-}$ treatment or (in mM): 2.5 NO_3_-N, 1.8 K, 0.6 Ca, 0.5 Mg, 0.5 S, and 0.5 P for the 2.5 mM ${\text{NO}}_{3}^{-}$ treatment. Both treatment solutions contained (in μM): 2 Mn, 2 Zn, 25 B, 0.5 Cu, 0.5 Mo, 100 Fe (as FeEDTA and FeEDDHA). Iron was supplemented twice weekly with the addition of Fe(NH_4_)_2_(SO_4_)_2_.6H_2_O (8 mg. l^−1^). Solution pH was maintained between 5.9 and 6.1. ${\text{NO}}_{3}^{-}$ was monitored using a ${\text{NO}}_{3}^{-}$ electrode (TPS, Springwood, Australia) and maintained at the target concentration ±10%. Other nutrients were monitored using an inductively coupled plasma optical emission spectrometer (ICP-OES: ARL 3580 B, ARL, Lausanne, Switzerland) and showed limited depletion between weekly solution changes. Plants were grown for 21 d prior to harvest.

### Flux measurement

At harvest, plants were transferred to a separate controlled environment room with equivalent growth conditions and solutions. Nitrate uptake capacity was measured as a short term (10 min) unidirectional flux measurement from a 200 μM ${\text{NO}}_{3}^{-}$ solution containing ^15^N labeled ${\text{NO}}_{3}^{-}$ (^15^N 10%) using the method described by Garnett et al. (2013) which was based on methods described in Kronzucker et al. (1995). After drying at 65°C for 7 days, total N, and ^15^N in the plant samples were determined with an isotope ratio mass spectrometer (Sercon, Cheshire, UK). The unidirectional ${\text{NO}}_{3}^{-}$ influx measured in this way is thought to reflect the uptake capacity of the plant at that point in time (Garnett et al., 2013). To measure the ${\text{NH}}_{4}^{+}$ uptake capacity the same protocol was followed but the fluxes were measured with 200 μM ${\text{NH}}_{4}^{+}$.

### Real-time quantitative PCR (Q-PCR)

On the same day the flux measurements were made, root material was harvested between 5 and 7 h after the start of the light period. The whole root was excised and snap-frozen in liquid N and stored at −80°C. RNA extraction and cDNA synthesis of these samples was carried out as described in Garnett et al. (2013) using the RNeasy Plant Mini Kit (Qiagen, Hilden, Germany) and SuperScriptIII reverse transcriptase (Invitrogen, Carlsbad, CA, USA). Real-time quantitative PCR (Q-PCR) and normalization was carried out as outlined in Burton et al. (2008). Four control genes were used in the normalization (*ZmGaPDh, ZmActin, ZmTubulin*, and *ZmElF1).* All primer sequences and Q-PCR product information for control genes and *NRT* and *AMT* genes can be found in Supporting Information Supplementary Table S2.

### Statistical analyses

A linear mixed model (LMM) procedure using ASReml-R package was used to analyse the phenotypic data (Butler et al., 2010; R Core Development Team, 2012). The fixed effects were taken to be the genotypes and ${\text{NO}}_{3}^{-}$ concentration (0.5 mM ${\text{NO}}_{3}^{-}$ or 2.5 mM ${\text{NO}}_{3}^{-}$). The random effect was taken to be the experiment number (a factor 1–7) in order to capture the variability of each experiment. The predicted values of each genotype in the two N concentrations for shoot dry weight, root dry weight, root to shoot ratio, N uptake, N uptake per unit root, shoot percentage of N, ${\text{NO}}_{3}^{-}$ uptake capacity, and ${\text{NH}}_{4}^{+}$ uptake capacity were obtained from the fitted models. The least significant difference (LSD) procedure was used to determine if the difference between the two N treatments is significant for each genotype at the 0.05 significance level for each trait. The percentages of explained variation attributed to genotypes, N concentrations, and their interaction were obtained from pseudo ANOVA tables using ASReml Wald test. Differences in transcript levels were analyzed using a Two way ANOVA with individual differences between treatments calculated using and uncorrected Fisher's LSD. Peasons correlation coefficients were calculated between the growth, flux and transcript measurements for the shortlisted lines.

## Results

### Growth and biomass

**Figure 1:**
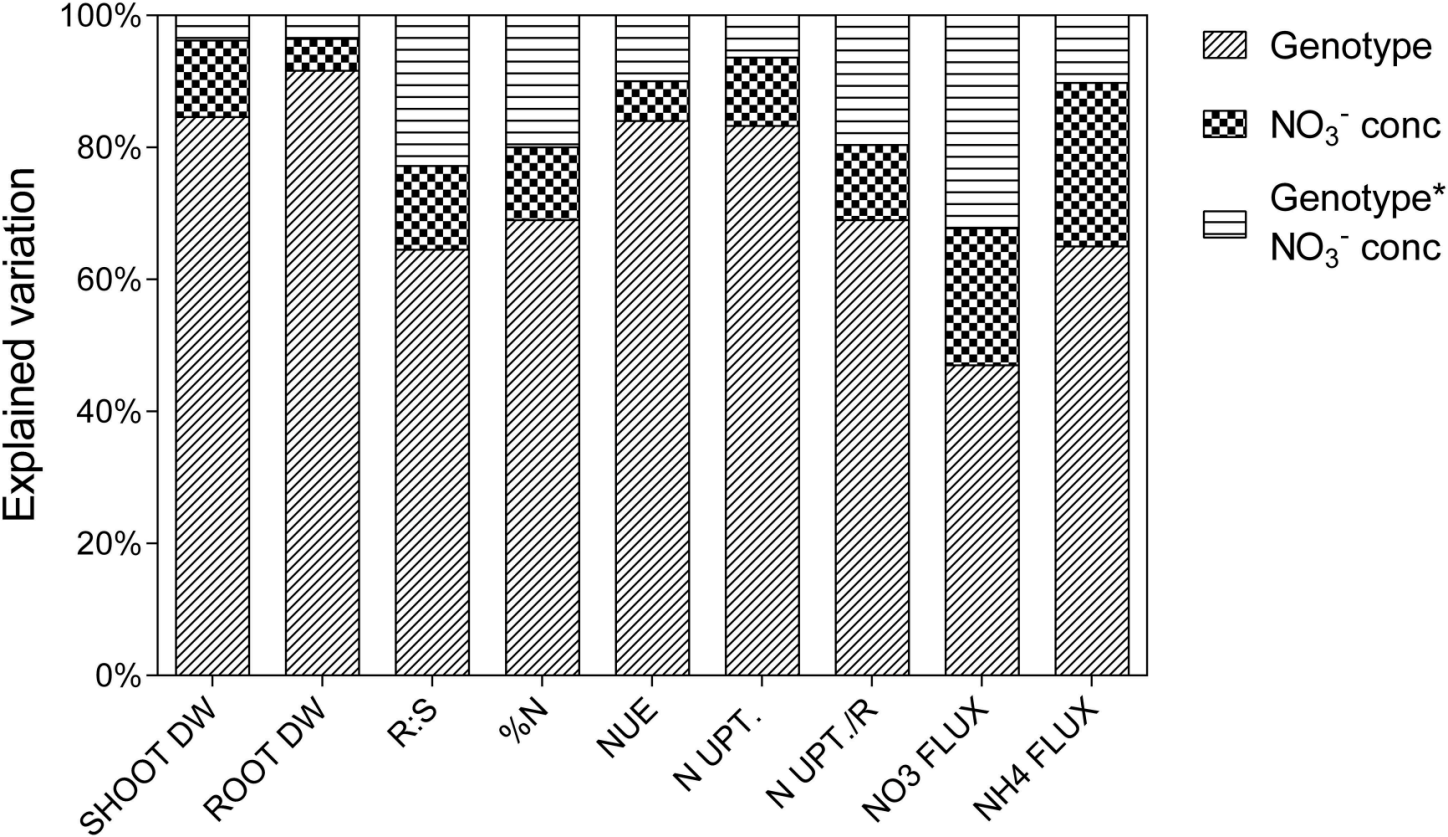
**Psuedo ANOVA results showing percentage of explained variation attributed to the treatments for each traits**. Shoot dry weight (SHOOT DW, g); root dry weight (ROOT DW, g); ratio of root to shoot dry weight (R:S); shoot % N (%N); vegetative NUE (NUE, %N/g DW); net N uptake to shoot (N UPT., gN); net N uptake to shoot relative to root dry weight (N UPT./R, gN.gDWroot^−1^); ${\text{NO}}_{3}^{-}$ flux (NO3 FLUX, nm.gDW^−1^.h^−1^); ${\text{NH}}_{4}^{+}$ flux (NH4 FLUX, nm.gDW^−1^.h^−1^).

**Figure 2:**
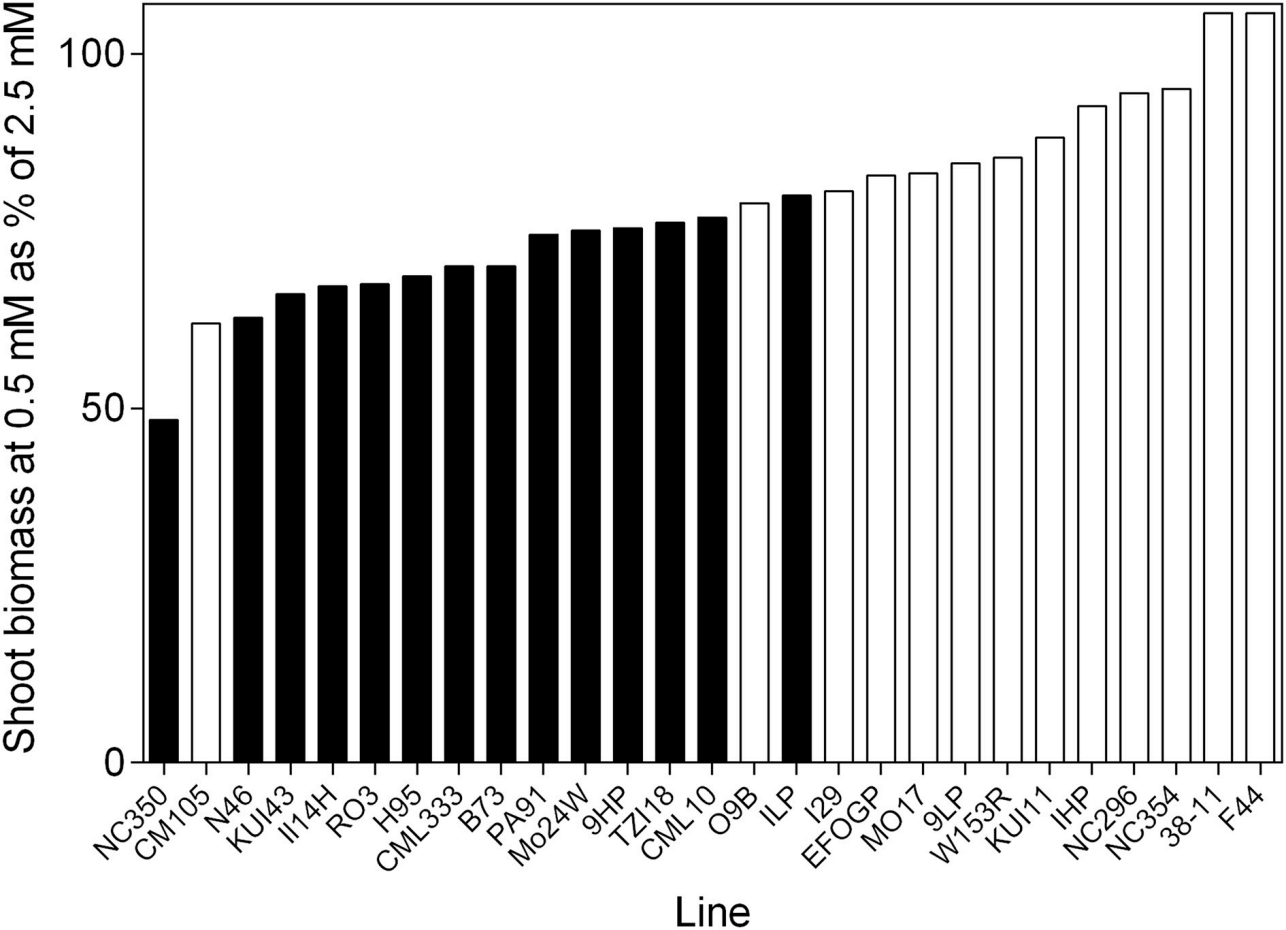
**The ability of 21 day old maize genotypes to retain biomass when grown at 0.5 mM nitrate with respect to growth at 2.5 mM nitrate**. Values are the predicted values of shoot biomass of plants grown at 0.5 mM ${\text{NO}}_{3}^{-}$ as a percentage of the predicted shoot biomass at 2.5 mM ${\text{NO}}_{3}^{-}$. Filled bars represent lines that are significantly smaller in the low N treatment at 0.05 significance level.

Percentage of variation explained by genotypes, ${\text{NO}}_{3}^{-}$ treatment or the interaction between these two factors as determined from ANOVA is presented in Figure 1. The majority of the explained variation is associated with genotypes, with considerably less being explained by the ${\text{NO}}_{3}^{-}$ treatment. The most substantial interaction term was for ${\text{NO}}_{3}^{-}$ uptake capacity, the genotypes differing greatly for this trait in response to ${\text{NO}}_{3}^{-}$ treatment. Although, the major source of variation in shoot biomass was the genotypes component there was still considerable variability in the response of germplasm to reduced ${\text{NO}}_{3}^{-}$ availability (Figure 2). The ratio of shoot biomass at 0.5 mM to that at 2.5 mM ${\text{NO}}_{3}^{-}$ ranged from genotypes for which biomass halved at reduced ${\text{NO}}_{3}^{-}$ to others that were able to maintain shoot biomass despite reduced ${\text{NO}}_{3}^{-}$ availability. All subsequent figures are displayed in terms of the ranking for biomass retention shown in Figure 2. In terms of the actual values of shoot biomass of plants grown at 0.5 mM with respect to growth at 2.5 mM ${\text{NO}}_{3}^{-}$, there was no consistent trend in the size of the plants, that is, neither the larger plants maintained biomass better at 0.5 mM ${\text{NO}}_{3}^{-}$ than smaller plants, nor vice versa. The general trend was that the plants with higher biomass tended to have higher biomass at both concentrations. However, the average size of plants in the mid-range of biomass retention (70–80%) was higher than at the extremes of either the high or the low % biomass retention.

**Figure 3:**
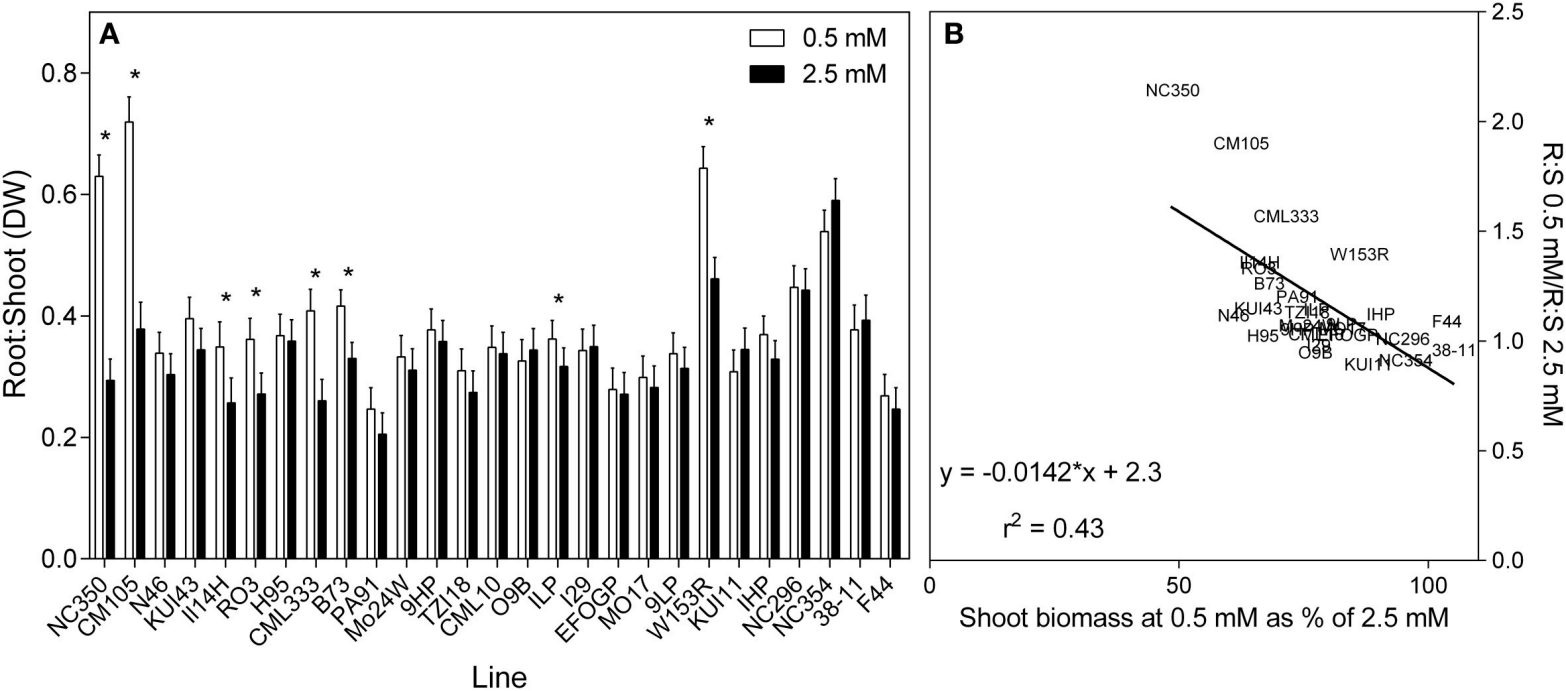
**Ratio of root to shoot dry weights of 21 day old maize genotypes grown at either 0.5 mM ${\text{NO}}_{3}^{-}$ or 2.5 mM ${\text{NO}}_{3}^{-}$(values are predicted values ± standard error, *n* = 8)**. **(A)** Genotypes are ordered from left to right according to their ability to retain biomass as shown in Figure 2 and **(B)** the scatterplot displaying the regression describing the relationship.

As shown in Figure 3A, most genotypes had similar root:shoot ratio (dry weight basis) of approximately 0.3. A few genotypes, however, for example NC296 and NC354, had higher ratios exceeding 0.4 but they tended to have smaller plants. In response to reduced ${\text{NO}}_{3}^{-}$, root:shoot ratio increased in genotypes at the lower end of % biomass retention. This can be seen more clearly in the scatterplot (Figure 3B), which shows plants able to maintain biomass at low N maintaining the same root:shoot under both N treatment conditions.

### N concentration

**Figure 4:**
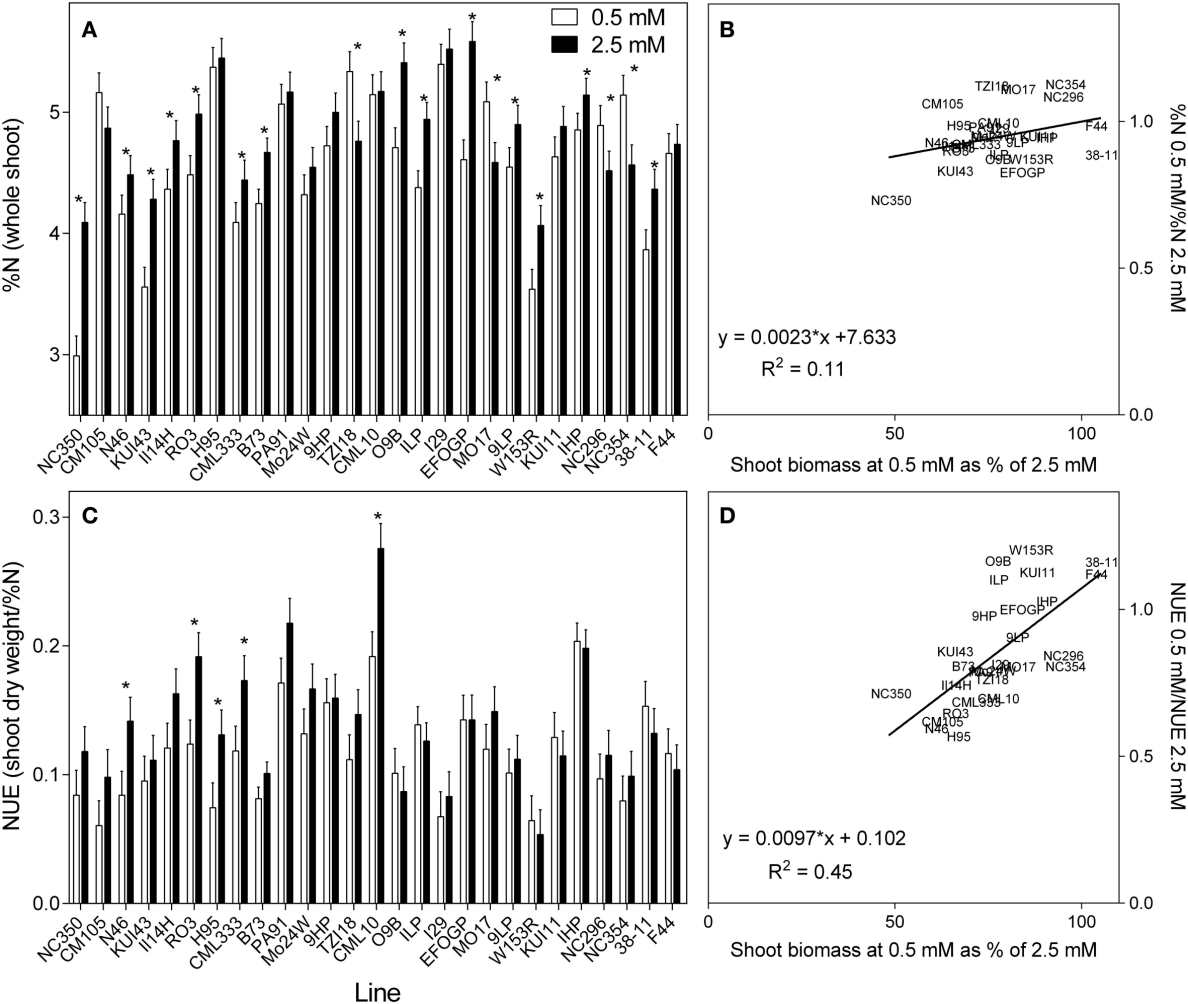
**The nitrogen (%) in the whole shoot (A), and NUE (shoot biomass per unit of N) (C) of 21 day old maize genotypes grown at either 0.5 mM nitrate or 2.5 mM nitrate**. Values are predicted values ± standard error (*n* = 8), ^*^ indicates those genotypes where %N and NUE were significantly different between the two growth conditions at the 0.05 significance level. Genotypes are ordered from left to right according to their ability to retain biomass as shown in Figure 2. Scatterplots **(B,D)** are the ratio of the values at each nitrate concentration plotted against ability to retain biomass.

The tissue concentrations of N varied much less than biomass; whereas the average reduction in shoot biomass at low N across all genotypes was 22%, the corresponding reduction in tissue %N was only 6.4%. These averaged data do, however, mask considerable variation. The shoot %N varied greatly between genotypes and treatments (Figure 4A), ranging from as high as 5.5% in the 2.5 mM treatment to as low as 3% in the 0.5 mM ${\text{NO}}_{3}^{-}$ treatment. There were significant differences between treatments (*p* < 0.05) and between genotypes (*p* < 0.001). As an average across all genotypes, a 6% reduction in %N was observed for the 0.5 mM treatment. However, this masks considerable variation between genotypes, the %N reduction being even greater in some genotypes, whilst some even had higher %N in the 0.5 mM ${\text{NO}}_{3}^{-}$ treatment. A consistent trend was that genotypes with higher %N at high N also had higher %N at low N. As shown in the associated scatterplot (Figure 4B), no clear correlation was observed between the ratio of %N at each concentration and the ability of a plant to maintain biomass. Given that plant growth at harvest was purely vegetative, NUE was calculated following Chardon et al. (2010) as the amount of shoot biomass per unit of N (shoot DM/%N) (Figure 4C). The NUE calculated in this way closely resembles the biomass results due mainly to the much greater variation in shoot growth than the %N. This can be seen clearly in the scatterplot, there being a close relationship between the NUE ratio and the ratio of shoot biomass (Figure 4D).

**Figure 5:**
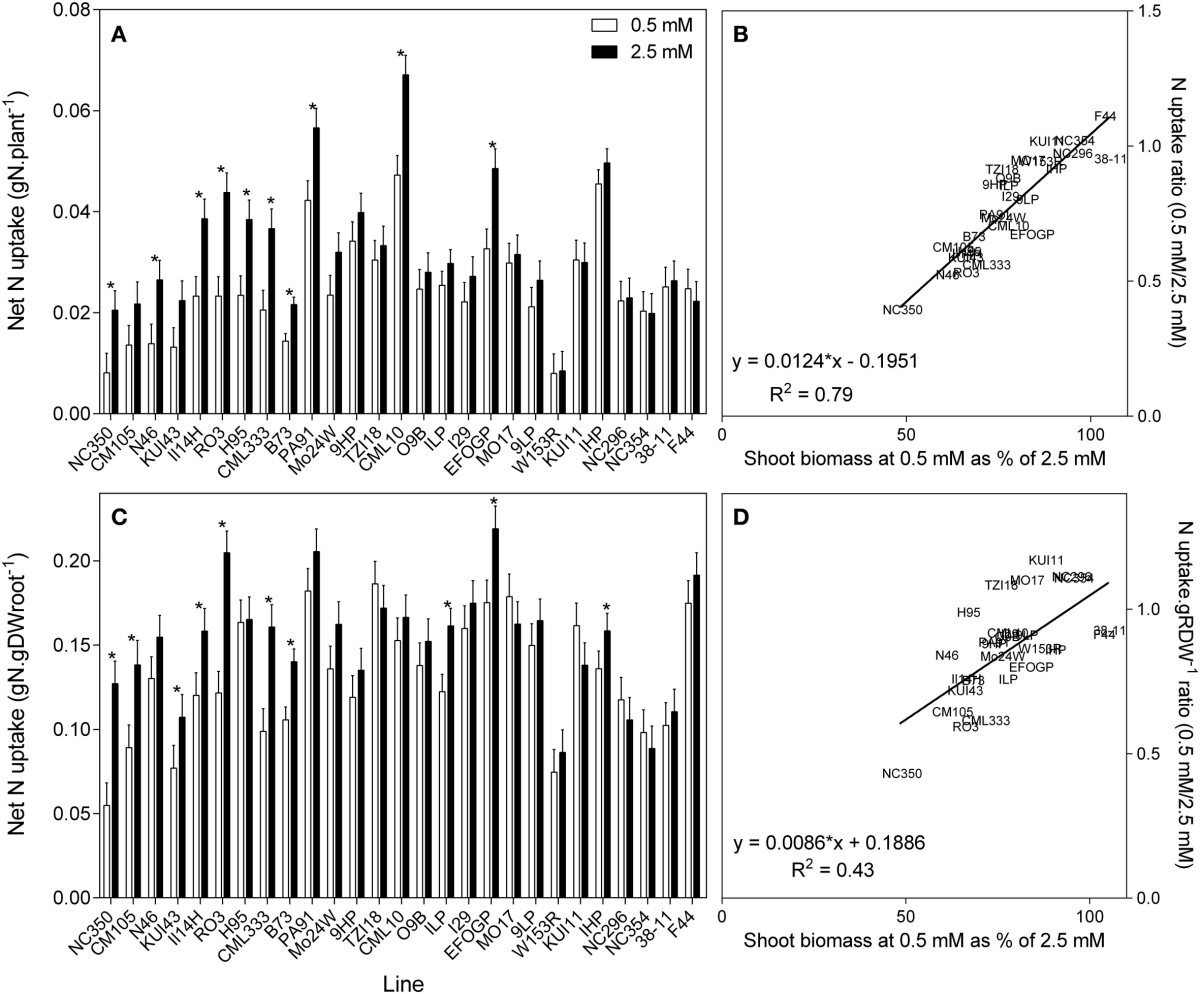
Net nitrogen uptake to the shoot (A), and net nitrogen uptake to the shoot relative to root size (C) of 21 day old maize genotypes grown at either 0.5 mM nitrate or 2.5 mM ${\text{NO}}_{3}^{-}$ (values are predicted values ± standard error (*n* = 8), ^*^ indicates those points that are significantly different between the two growth conditions at 0.05 significance level. Genotypes are ordered from left to right according to their ability to retain biomass as shown in Figure 2. Scatterplots **(B,D)** are the ratio of the values at each nitrate concentration plotted against ability to retain biomass.

It is possible to use the shoot %N to calculate the total net N uptake to the shoot (Figure 5A). There is a clear trend for those plants with reduced biomass with reduced ${\text{NO}}_{3}^{-}$ availability also having lower net N uptake. The reciprocal is that plants that maintained their biomass at low ${\text{NO}}_{3}^{-}$ succeeded in maintaining net N uptake despite the reduced ${\text{NO}}_{3}^{-}$ availability. When root size is taken into account and net N uptake is calculated per gram root weight, this relationship is less strong but still apparent (Figure 5C). The described relationships are clearer in the regressions between the ratio of N uptake and biomass retention (Figures 5B,D).

### Fluxes

The unidirectional influx of ${\text{NO}}_{3}^{-}$ and ${\text{NH}}_{4}^{+}$ was measured at a concentration of 200 μM as a means of providing a snapshot of the uptake capacity of the genotypes in response to N supply. At this concentration the high affinity transport system (HATS) ought to be saturated and the measured uptake capacity should approximate the maximum uptake capacity of the HATS (Siddiqi et al., 1990; Kronzucker et al., 1995; Reid, 1998; Garnett et al., 2003). Based on previous measurements with maize it was anticipated that there would be limited LATS contribution to the net uptake in the low ${\text{NO}}_{3}^{-}$ treatment and that the HATS would provide possibly 50% of net uptake in the high ${\text{NO}}_{3}^{-}$ treatment (Garnett et al., 2013).

**Figure 6:**
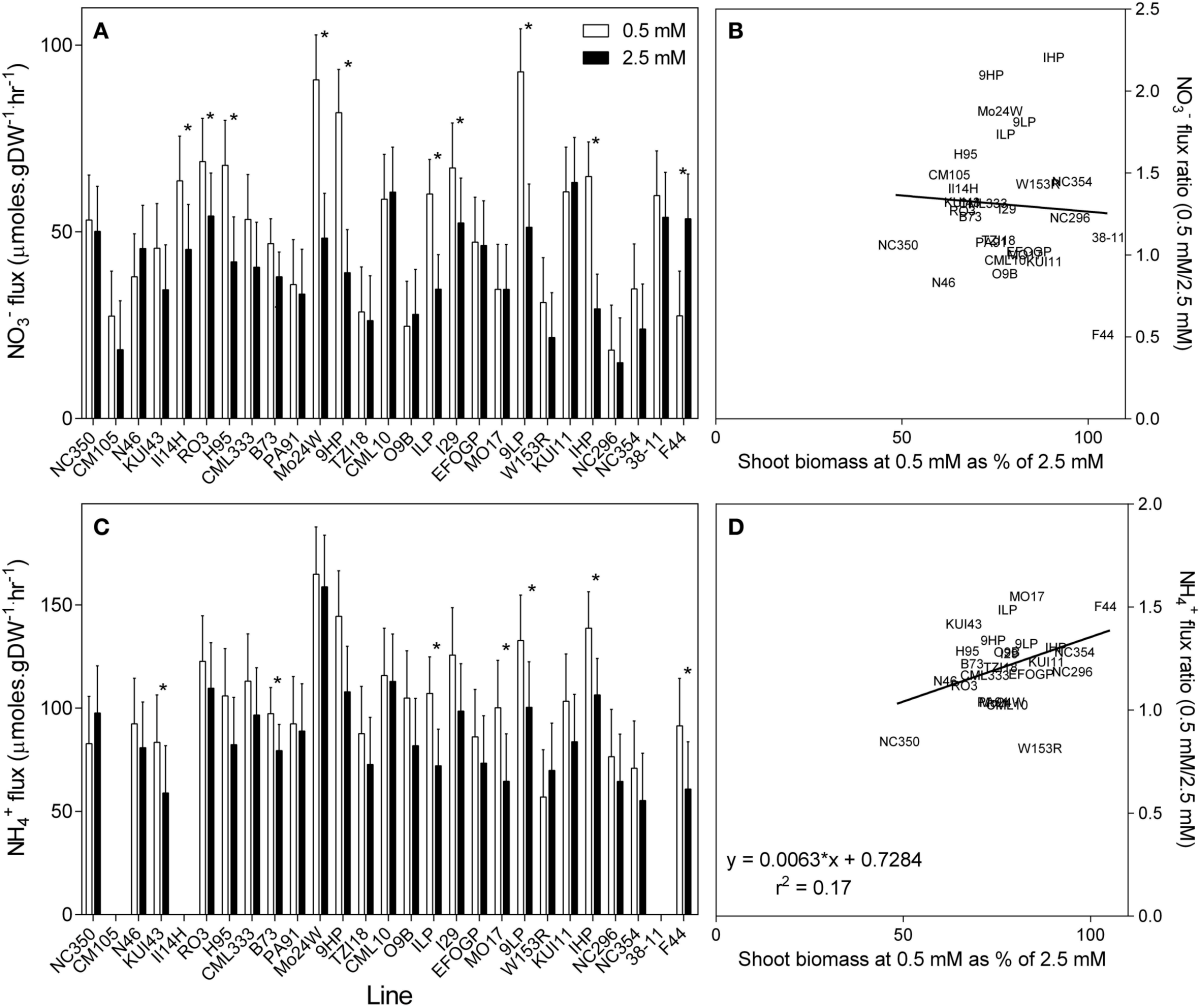
**Unidirectional influx of ${\text{NO}}_{3}^{-}$ (A) and ${\text{NH}}_{4}^{+}$ (C) into the roots of maize genotypes grown at either 0.5 mM or 2.5 mM ${\text{NO}}_{3}^{-}$**. Nitrate and ${\text{NH}}_{4}^{+}$ influx were measured using^15^N labeled ${\text{NO}}_{3}^{-}$ or ${\text{NH}}_{4}^{+}$ over a 10-min influx period from a 200 μM solution. Values are predicted values ± standard error (*n* = 4) ^*^ indicates those points that are significantly different between the two growth conditions at 0.05 significance level. Genotypes are ordered from left to right according to their ability to retain biomass as shown in Figure 2. Missing values for ${\text{NH}}_{4}^{+}$ flux are due to no measurement of ${\text{NH}}_{4}^{+}$ flux being made on these lines. Scatterplots **(B,D)** are the ratio of the values at each nitrate concentration plotted against their ability to retain biomass.

Nitrate flux was measured using 200 μM ^15^N ${\text{NO}}_{3}^{-}$ over a 10 min loading period (Garnett et al., 2013). Nitrate uptake capacity measured in this way is shown in Figure 6A. In general, the ${\text{NO}}_{3}^{-}$ uptake capacity of roots grown at 0.5 mM ${\text{NO}}_{3}^{-}$ was 30% higher than that measured in plants grown at 2.5 mM ${\text{NO}}_{3}^{-}$. However, this increase in ${\text{NO}}_{3}^{-}$ uptake capacity was not uniform, with some genotypes having a similar ${\text{NO}}_{3}^{-}$ uptake capacity whether grown at high or low ${\text{NO}}_{3}^{-}$.

**Figure 7:**
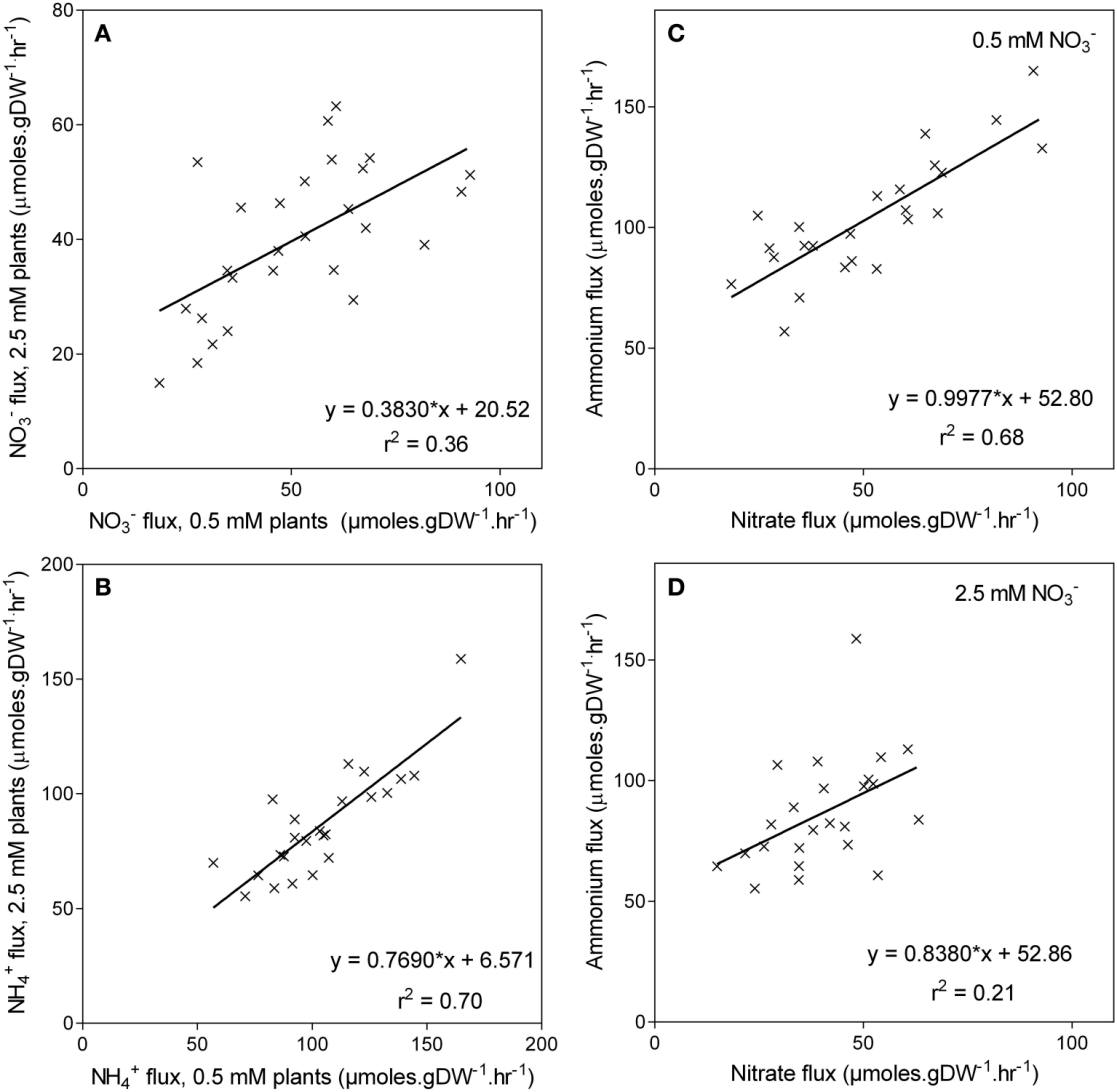
**Unidirectional influx of ${\text{NO}}_{3}^{-}$ and ${\text{NH}}_{4}^{+}$into the roots of maize genotypes grown at either 0.5 mM or 2.5 mM ${\text{NO}}_{3}^{-}$**. **Panels (A,B)** show the relationships between the fluxes of either ${\text{NO}}_{3}^{-}$ **(A)** or ${\text{NH}}_{4}^{+}$ **(B)** for each ${\text{NO}}_{3}^{-}$ treatment; the relationship between the fluxes for each ${\text{NO}}_{3}^{-}$ treatment is shown in **Panels (C,D)**. Nitrate and ${\text{NH}}_{4}^{+}$ influx were measured using^15^N labeled ${\text{NO}}_{3}^{-}$ or ${\text{NH}}_{4}^{+}$ over a 10-min influx period from a 200 μM solution.

High-affinity ${\text{NH}}_{4}^{+}$ uptake capacity was also measured as a unidirectional flux of 200 μM ^15^N ${\text{NH}}_{4}^{+}$. Averaged across the genotypes the ${\text{NH}}_{4}^{+}$ uptake capacity was almost double the ${\text{NO}}_{3}^{-}$ uptake capacity (Figure 6C). As observed with ${\text{NO}}_{3}^{-}$, the ${\text{NH}}_{4}^{+}$ uptake capacity was also higher in the low ${\text{NO}}_{3}^{-}$ grown plants, being on average 22% higher in plants grown at 0.5 mM ${\text{NO}}_{3}^{-}$ compared with those grown at 2.5 mM ${\text{NO}}_{3}^{-}$. Although, ${\text{NO}}_{3}^{-}$ and ${\text{NH}}_{4}^{+}$ uptake capacity was elevated in low ${\text{NO}}_{3}^{-}$ grown plants, no correlation was found between uptake capacity and the ability of these genotypes to retain biomass in low ${\text{NO}}_{3}^{-}$ (Figures 6B,D). Likewise, genotypes with higher ${\text{NH}}_{4}^{+}$ uptake capacity when grown in 0.5 mM ${\text{NO}}_{3}^{-}$ had higher ${\text{NH}}_{4}^{+}$ uptake capacity when grown at 2.5 mM ${\text{NO}}_{3}^{-}$ (*r*^2^ = 0.70, *p* < 0.001), although this relationship was not as pronounced (*r*^2^ = 0.36) in the ${\text{NO}}_{3}^{-}$ uptake capacity (Figures 7A,B). There was also a correlation (*r*^2^ = 0.68, *p* < 0.001) between ${\text{NH}}_{4}^{+}$ and ${\text{NO}}_{3}^{-}$ uptake capacity in the 0.5 mM ${\text{NO}}_{3}^{-}$ grown plants, in that the genotypes with high ${\text{NO}}_{3}^{-}$ uptake capacity also had a high ${\text{NH}}_{4}^{+}$ uptake capacity (Figure 7C). However, this relationship was no longer apparent in the high ${\text{NO}}_{3}^{-}$ treatment (*r*^2^ = 0.21) (Figure 7D).

### Transporter transcript levels

**Figure 8:**
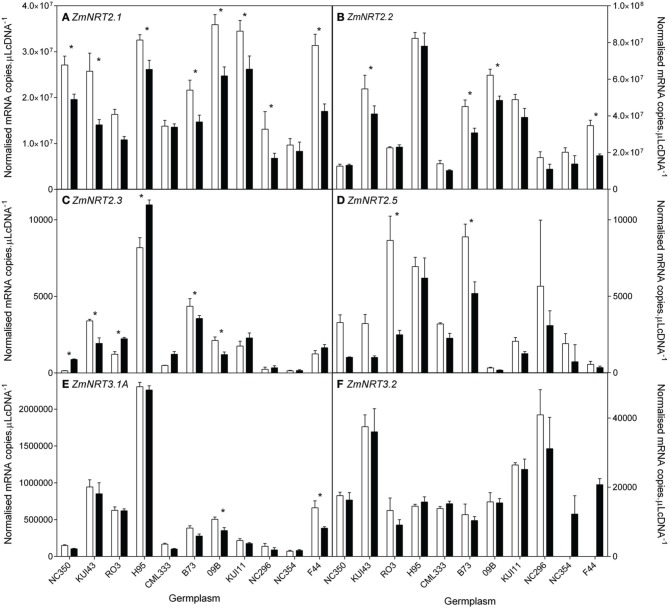
**Root transcript levels of putative high affinity (NRT2) ${\text{NO}}_{3}^{-}$ transporters (A–D) and two putative NRT3 (E,F) proteins in diverse maize genotypes grown in nutrient solution containing either 0.5 mM (open bars) or 2.5 mM (closed bars) ${\text{NO}}_{3}^{-}$**. Each data point is normalized against control genes (*ZmGaPDh, ZmActin, ZmTubulin*, and *ZmElF1*). Values are means ± SEM (*n* = 4), ^*^ indicates those points that are significantly different between the two growth conditions at 0.05 significance level. Genotypes are ordered from left to right according to their ability to retain biomass as shown in Figure 2.

**Figure 9:**
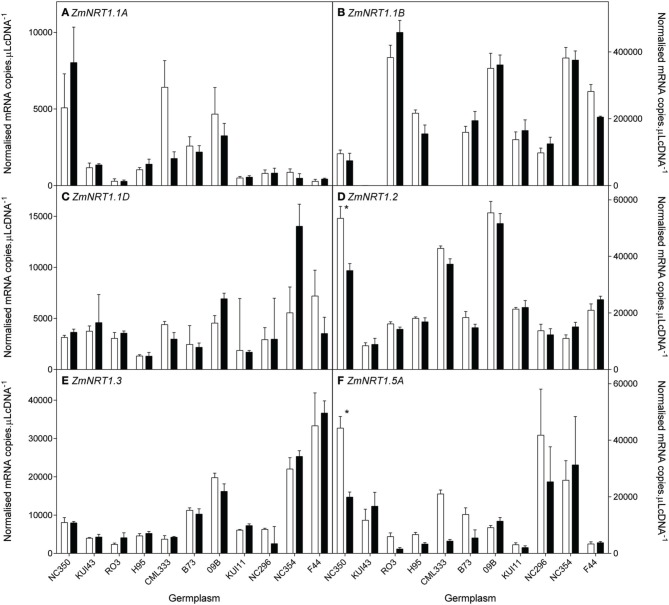
**Root transcript levels of various putative low affinity (NRT1) ${\text{NO}}_{3}^{-}$ transporters (A–F) in diverse maize genotypes grown in nutrient solution containing either 0.5 mM (open bars) or 2.5 mM (closed bars) ${\text{NO}}_{3}^{-}$**. Each data point is normalized against control genes (*ZmGaPDh, ZmActin, ZmTubulin*, and *ZmElF1*). Values are means ± SEM (*n* = 4), ^*^ indicates those points that are significantly different between the two growth conditions at 0.05 significance level. Genotypes are ordered from left to right according to their ability to retain biomass as shown in Figure 2.

**Figure 10:**
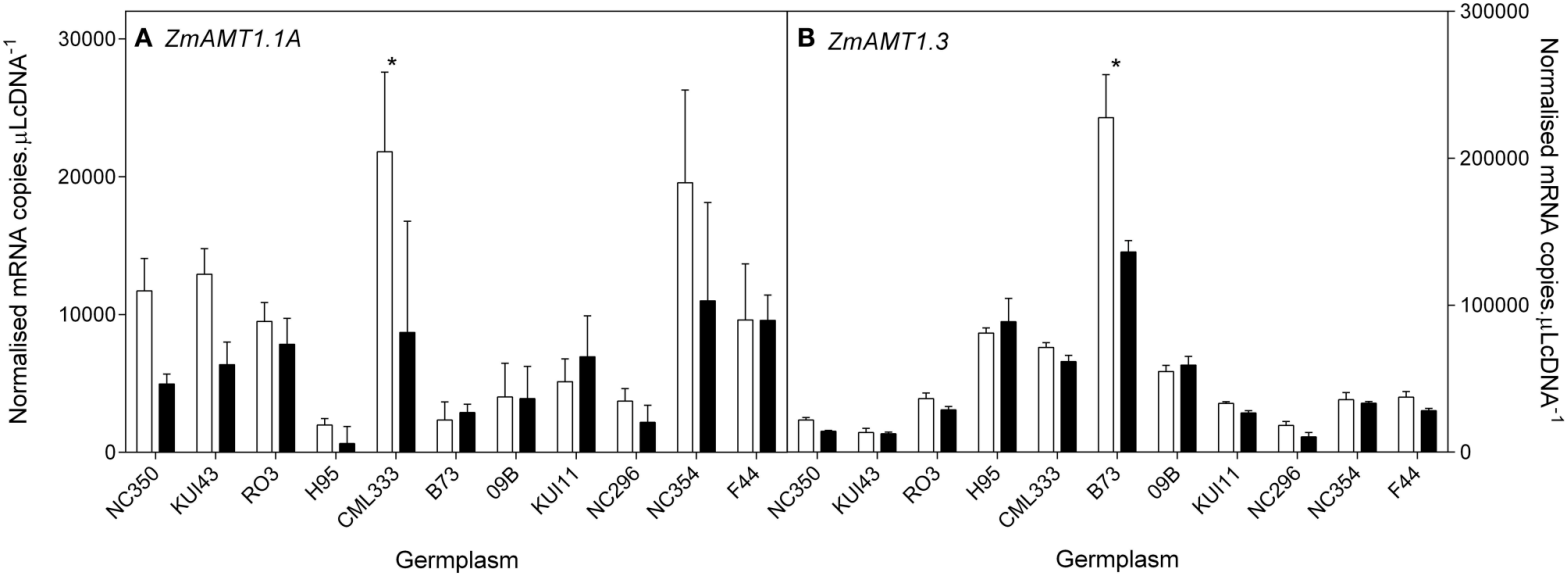
**Root transcript levels of two putative AMT ${\text{NH}}_{4}^{+}$ transporters (A,B) in diverse maize genotypes grown in nutrient solution containing either 0.5 mM (open bars) or 2.5 mM (closed bars) ${\text{NO}}_{3}^{-}$**. Each data point is normalized against control genes (*ZmGaPDh, ZmActin, ZmTubulin*, and *ZmElF1*). Values are means ± SEM (*n* = 4), ^*^ indicates those points that are significantly different between the two growth conditions at 0.05 significance level. Genotypes are ordered from left to right according to their ability to retain biomass as shown in Figure 2.

In order to understand the observed differences in uptake capacity, ten of the genotypes were selected for measurement of the root transcript levels of putative high and low affinity (*NRT1, NRT2*, and *NRT3*) ${\text{NO}}_{3}^{-}$ transporter genes and putative high affinity *AMT* ${\text{NH}}_{4}^{+}$ transporter genes. The 10 genotypes were selected to include a wide range of biomass retention in response to ${\text{NO}}_{3}^{-}$ supply (Figure 2). At the whole root level, transcript levels of the ${\text{NO}}_{3}^{-}$ HATS genes (*ZmNRT2.1, ZmNRT2.2*) and *ZmNRT3.1* dominated the total RNA pool when compared with the putative ${\text{NO}}_{3}^{-}$ LATS genes *(ZmNRT1.1a, ZmNRT1.1b, ZmNRT1.2, ZmNRT1.5A)* or the *ZmAMT*s (Figures 8–10). The transcript abundance of the other putative ${\text{NO}}_{3}^{-}$ transporter genes was significantly lower. Considerable variability existed between genotypes in the root transcript levels of all the transporter genes examined (Figures 8–10). The transcript levels of *ZmNRT2.1, ZmNRT2.2*, and *ZmNRT2.3* showed the largest response to low ${\text{NO}}_{3}^{-}$.

**Figure 11:**
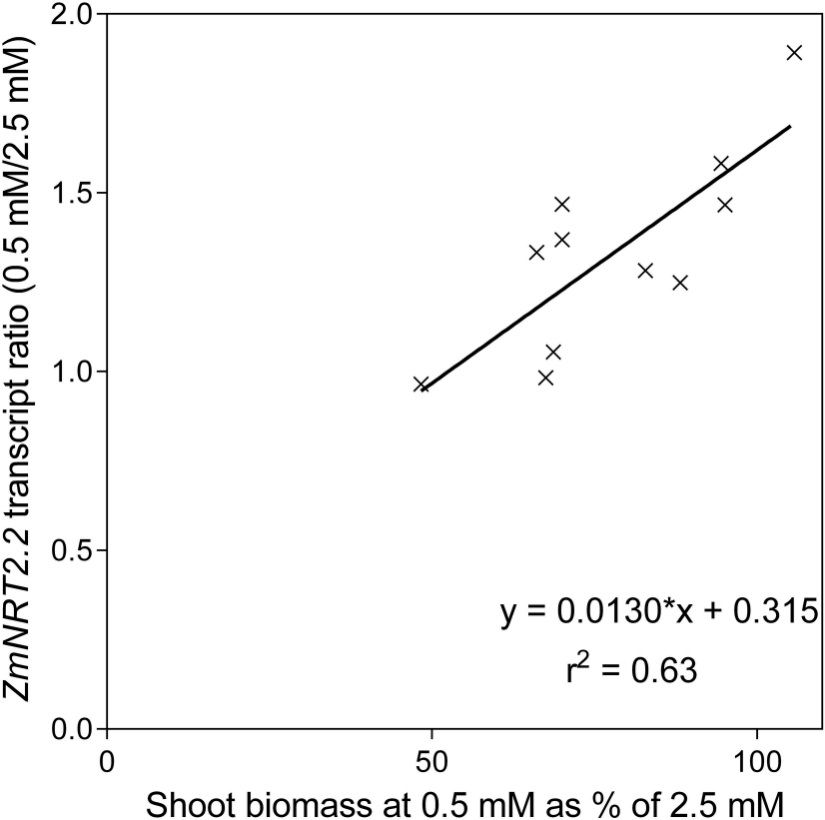
**The relationship between the ratio of *ZmNRT2.2* transcript levels and the ability to maintain shoot biomass between nutrient solution containing either 0.5 mM or 2.5 mM ${\text{NO}}_{3}^{-}$**.

None of the transporter transcript levels showed good correlation with flux parameters but the ratio of *ZmNRT2.2* transcript levels between the two N levels was positively correlated with the maintenance of shoot biomass at reduced N (Figure 11, Supplementary Data Sheet 1). There were strong correlations between transcript levels of *ZmNRT2.2* and *2.3*, and *ZmNRT3.1a*.

## Discussion

Considerable variability was found among the genotypes in their growth response to N supply. Given that limitation of shoot growth ranged from no restriction up to a 50% reduction (Figure 2), it is apparent that the concentrations of 0.5 and 2.5 mM ${\text{NO}}_{3}^{-}$ were appropriate for separating the genotypes on the basis of N response. Although the biomass response to N supply was significant, the underlying biomass and N transport characteristics showed less variation, with generally only a third of the tested lines showing significant differences between the N treatments. Certain genotypes were found to have a decreased %N despite being grown in the higher N treatment, this possibly being evidence of a growth dilution (Greenwood et al., 1990); most genotypes had decreased %N with reduced N (average across genotypes: 0.5 mM, 4.57%; 2.5 mM, 4.81%. Despite the significant restriction of shoot growth, few of the genotypes had %N levels that would correspond to N deficiency (Reuter and Robinson, 1997).

Large differences were found in vegetative NUE (Chardon et al., 2010), measured as the shoot vegetative biomass per unit of tissue N (Figure 4C). Vegetative NUE estimates are affected heavily by shoot biomass, which in the current study changed more dramatically than tissue N. Similarly, no correlation was found between %N and the ability of plants to maintain biomass at reduced N. Together, the %N and NUE results suggest that none of the genotypes were able to grow unimpeded in the reduced ${\text{NO}}_{3}^{-}$ concentration by simply increasing the efficiency of N use. Rather, the NUE decreased with reduced ${\text{NO}}_{3}^{-}$ in plants less able to maintain biomass at 0.5 mM ${\text{NO}}_{3}^{-}$. In light of this finding combined with the fact that vegetative NUE may have no relationship to the grain NUE (grain yield per unit of N) (Moll et al., 1982; Dhugga and Waines, 1989; Good et al., 2004), it is suggested that at the early growth stage examined here, vegetative NUE (or N utilization efficiency) may not be an important component in the response of a plant to low N.

The classic response of a plant to low N is to first increase the N absorption potential of the roots and then increase root size relative to shoot size in order to capture more N by exploring a greater volume of soil (Chapin, 1991). Increasing root size relative to shoot size in response to low N is not an ideal strategy as it diverts carbon from the shoots thus restricting further carbon capture. In this study genotypes maintaining biomass at reduced N were able to maintain N uptake, and did so without any change in root biomass relative to shoot biomass, perhaps reflecting the importance of maintaining carbon capture.

The uptake capacity for ${\text{NH}}_{4}^{+}$ was much higher than that for ${\text{NO}}_{3}^{-}$, as has been demonstrated for monocots and dicots (Clarkson and Warner, 1979; Clarkson et al., 1986; Kronzucker et al., 1995, 1996; Garnett et al., 2003). This may simply reflect the adaptability of maize roots and roots in general to acquire ${\text{NH}}_{4}^{+}$ at the low concentrations commonly found in agricultural soils; these soils generally contain ${\text{NH}}_{4}^{+}$ at only 10% of the ${\text{NO}}_{3}^{-}$ concentration (Wolt, 1994; Miller et al., 2007). Regardless of the absolute differences, for plants grown on reduced ${\text{NO}}_{3}^{-}$ supply, increased uptake capacity was measured for ${\text{NO}}_{3}^{-}$ and, to a lesser extent, ${\text{NH}}_{4}^{+}$. Although, the average response was an increase in uptake capacity at low N, the interaction term in the ANOVA emphasizes the considerable differences in response between genotypes.

The proposition, based on the plant response by Chapin (1991) outlined earlier, that plants maintaining N uptake with reduced ${\text{NO}}_{3}^{-}$ supply would show an increased uptake capacity would seem reasonable. However, although there was an increase in ${\text{NO}}_{3}^{-}$ and ${\text{NH}}_{4}^{+}$ uptake capacity with reduced N, there was no association between uptake capacity and either total N uptake or the ability to maintain biomass at reduced N. Recently, it has been shown that under steady state N supply there was substantial variation in ${\text{NO}}_{3}^{-}$ uptake capacity during vegetative growth of maize (Garnett et al., 2013). A similar variation in the genotypes measured here could well explain the disparity observed between N uptake capacity and net N uptake.

Transcripts of a total of 10 *NRT1*, 4 *NRT2*, and 3 *NRT3* genes were quantified in root tissue and only those with quantifiable transcripts are presented here. Of the transcripts within the total RNA pool, *ZmNRT2.1* and *ZmNRT2.2* were the most abundant. The transcriptional response of the putative ${\text{NO}}_{3}^{-}$ and ${\text{NH}}_{4}^{+}$ transporter genes to reduced N supply is indicative of their roles. The observed increase in uptake capacity with reduced N was reflected in an increase in transcript levels of certain of the putative transporter genes, namely *ZmNRT2.1, ZmNRT2.2*, and *ZmNRT2.3.* In a lifecycle study with dwarf maize, Garnett et al. (2013) found that similar genes were most transcriptionally responsive in response to N supply and demand. In a study comparing three maize lines with differing NUE, El-Kereamy et al. (2011) found that, as in this study, *ZmNRT2.3* root transcript levels were higher with N limitation. The most consistent transcript level increase with low ${\text{NO}}_{3}^{-}$ was observed with *ZmNRT2.1*, and together with its dominance of the total RNA pool, this suggests that NRT2.1 may be responsible for a significant proportion of ${\text{NO}}_{3}^{-}$ uptake, as orthologous transporters are in Arabidopsis and other plants (Tsay et al., 2007; Garnett et al., 2009; Dechorgnat et al., 2011). That the ratio of *ZmNRT2.2* transcript levels between the two N treatments was correlated with the ability to maintain shoot biomass, implies that this transporter plays an important role in the response to low N, and that this transporter is part of the mechanism by which some plants are better able to cope with reduced N supply. That the ZmNRT2.2 transcript levels did not correlate with uptake capacity or net uptake may again, as with flux capacity and net uptake, be related to the temporal variability observed in flux capacity and ${\text{NO}}_{3}^{-}$ transporter transcript levels observed previously in maize (Garnett et al., 2013). AtNRT3.1 has been found to be essential for function of AtNRT 2s (Okamoto et al., 2006; Wirth et al., 2007; Yong et al., 2010; Kotur et al., 2012). Surprisingly, although *ZmNRT3.1a* was similar to *ZmNRT2.1* and *2.2* in transcript abundance, it did not show the treatment differences the *ZmNRT2s* did. Regardless, the correlations between *ZmNRT3.1a* and both *ZmNRT2.2* and *2.3* supports the inovlement of ZmNRT3.1a and the ZmNRTs. Gu et al. (2013) found that *AMT1.1A* and *AMT1.3* transcript levels were not upregulated by N demand but rather were induced by ${\text{NH}}_{4}^{+}$ and this appears to be the case also in maize.

The current study has demonstrated that certain genotypes are able to maintain N uptake under conditions of reduced N availability; gaining knowledge of the basis of the higher N uptake efficiency of these genotypes will be an important step toward understanding the underlying biology of NUpE. As shown by Debruin et al. (2013), an efficient N uptake system is essential for grain development as up to 60–70% of grain N in maize is absorbed after flowering. The results obtained are consistent with genotypes being better able to cope with a reduction in N availability, as distinct from N deficiency, and being better able to increase ${\text{NO}}_{3}^{-}$ uptake without increasing carbon allocation to the roots. This would confer on these genotypes a competitive advantage as increasing carbon allocation to the roots could potentially restrict shoot growth and carbon fixation.

The transcriptional response of the ${\text{NO}}_{3}^{-}$ and ${\text{NH}}_{4}^{+}$ transporter genes to N supply assists in clarifying the roles of these genes and identifying those which may be important in developing superior NUE genotypes. Further, research will focus on a subset of these lines to discover the mechanism by which plants maintaining uptake without changing root size, focussing on the roles of the ZmNRT2s, in particularly ZmNRT2.2.

## Author contributions

TG, SC, VC, KD, AR, MT, and BK conceived and designed the experiments. TG, DP, SC, and VC performed the experiments and sample analysis. TG, DP, SC, VC, HR, AR, KD, MT, and BK acquired and analyzed the data. TG, DP, VC, SC, HR, AR, KD, MT, and BK wrote the manuscript.

## Funding

This project was funded by the Australian Centre for Plant Functional Genomics, DuPont Pioneer, and an Australian Research Council Linkage Grant (LP0776635) to BK, MT (University of Adelaide) AR, and KD (DuPont Pioneer).

### Conflict of interest statement

The authors declare that the research was conducted in the absence of any commercial or financial relationships that could be construed as a potential conflict of interest.
