# Supplementary material for: Variation for N Uptake System in Maize: Genotypic Response to N Supply
Source: Front Plant Sci. 2015 Nov 9;6:936. doi: 10.3389/fpls.2015.00936 (PMC4637428; doi:10.3389/fpls.2015.00936)
Supplement: Supplementary file 2 [file DataSheet2.DOCX]

***Supplementary Material***

**Genotypic diversity in the response of the maize nitrogen uptake system to nitrogen supply**

**Trevor Garnett*, Darren Plett, Vanessa Conn, Simon Conn, Huwaida Rabie, Antoni Rafalski, Kanwarpal Dhugga, Mark Tester, Brent N. Kaiser**

*** Correspondence:** Corresponding Author: trevor.garnett@adelaide.edu.au

**Supplementary Table 1.** Name and background of the maize lines used in the study.

| **Line** | **Background** | **Line** | **Background** |
| --- | --- | --- | --- |
| Mo24W | Mixed | NC354 | Tropical Stiff Stalk |
| YKI43 | Mixed | TZI18 | Tropical Stiff Stalk |
| 38-11 | Non Stiff Stalk | YKI11 | Tropical Stiff Stalk |
| F44 | Non Stiff Stalk | O9B | Stiff Stalk |
| H95 | Non Stiff Stalk | EFOGP | Stiff Stalk |
| MO17 | Non Stiff Stalk | N46 | Non Stiff Stalk |
| PA91 | Non Stiff Stalk | RO3 | Non Stiff Stalk |
| W153R | Non Stiff Stalk | IHP | Sweetcorn |
| B73 | Stiff Stalk | Il14H | Sweetcorn |
| CM105 | Tropical Stiff Stalk | 9HP | Sweetcorn |
| CML10 | Tropical Stiff Stalk | 9LP | Sweetcorn |
| CML333 | Tropical Stiff Stalk | ILP | Sweetcorn |
| NC296 | Tropical Stiff Stalk | I29 | Popcorn |
| NC350 | Tropical Stiff Stalk |  |  |
|  |  |  |  |
|  |  |  |  |

**Supplementary Table 2.** Q-PCR primers for assay of maize gene expression are listed along with the Q-PCR product size (bp).

| Gene | Gene ID | Forward Primer(5'->3') | Reverse Primer (5'->3') | Q-PCR Product size (bp) |
| --- | --- | --- | --- | --- |
| *ZmNRT1.1A* | GRMZM2G086496 | CCTCCAGCAAGAAGAGCAAG | GACACCGAGAAGGTGGTCA | 238 |
| *ZmNRT1.1B* | GRMZM2G161459 | GTCATCAGCGCCATCAACCT | GGGTCACACCGTGTGCCAAA | 282 |
| *ZmNRT1.1D* | GMRZM2G161483 | CAGCACCGCCATCGTCAG | GCCAGCAGCCAATAGAACTTG | 114 |
| *ZmNRT1.2* | GRMZM2G137421 | GGTGCTGCCCATCTTCTTGT | ATGATGTGGTCGTAGACGGG | 186 |
| *ZmNRT1.3* | GMRZM2G176253 | CGCCGTCTTCGTCGTCTTC | AAGTCGTCCATCTCCTTGTGC | 102 |
| *ZmNRT1.5A* | GRMZM2G044851 | CGTATGTTGTTCTTGTCTTCTTG | GTGCTATCGTCGTCAATGG | 104 |
| *ZmNRT2.1* | GRMZM2G010280 | CGACGAGAAGAGCAAGGGACT | GGCATATTCGTACATACAAAGAGGT | 183 |
| *ZmNRT2.2* | GRMZM2G010251 | CGACGAGAAGAGCAAGGGACT | AGGTGAACATGGATGATGGAT | 166 |
| *ZmNRT2.3* | GRMZM2G163866 | AGGAAGGGCATCGAGAACAT | CTTGCGCTGTGACGGCCTAC | 179 |
| *ZmNRT2.5* | GMRZM2G455124 | GCATCGTCCCGTTCGTCTC | CCGTCTCCGTCTTGTACTTGG | 129 |
| *ZmNRT3.1A* | GRMZM2G179294 | GCATCCACGCCTCTCTCAAG | TCAGCAACGACAGCCACTCAT | 177 |
| *ZmNRT3.2* | GMRZM2G808737 | GTCGCTCATTCCTCGGTGTC | TTGATGTTGCCTTGTTCGTTCC | 96 |
| *ZmAMT1.1A* | GRMZM2G175140 | CCAGCAGCCAGGTGTAAAA | CGACTCCCAAGTAGCCAAG | 161 |
| *ZmAMT1.3* | GRMZM2G028736 | TGGACTCGACGTACCTGCTCT | AAGAAGTGCTTGCCGATGAAG | 217 |
| *ZmGaPDh* | GRMZM2G077927 | GACAGCAGGTCGAGCATCTTC | GTCGACGACGCGGTTGCTGTA | 114 |
| *ZmActin* | GRMZM2G126069 | CCAATTCCTGAAGATGAGTCT | TGGTAGCCAACCAAAAACAGT | 156 |
| *ZmTubulin* | GRMZM2G152466 | GAGGACGGCGACGAGGGTGAC | CAAAGCGGGGGAATAAAGTCT | 186 |
| *ZmElF1* | GRMZM2G154218 | GCCGCCAAGAAGAAATGATGC | CGCCAAAAGGAGAAATACAAG | 220 |

**Supplementary Figure 1.** Shoot dry weight of 21 day old maize genotypes grown at either 0.5 mM NO_3_^-^ or 2.5 mM NO_3_^-^ . Values are predicted values ± standard error (n=8), * indicates those genotypes where the dry weight was significantly different between the two growth conditions at 0.05 significance level. Genotypes are ordered from left to right according to their ability to retain biomass as shown in Fig. 2.





**Supplementary Spreadsheet 1.** Pearson’s correlation coefficients for the subset of lines used for the transporter transcript analysis. Positive correlation are highlighted
